# Effectiveness of various types of coating materials applied in reinforced concrete exposed to freeze–thaw cycles and chlorides

**DOI:** 10.1038/s41598-023-40203-8

**Published:** 2023-08-10

**Authors:** Ginneth Patricia Millán Ramírez, Hubert Byliński, Maciej Niedostatkiewicz

**Affiliations:** Department of Engineering Structures, Faculty of Civil and Environmental Engineering, Gdansk University of Technology, 11/12 Narutowicza Street, 80-226 Gdańsk, Poland

**Keywords:** Civil engineering, Mechanical properties

## Abstract

This study assesses the durability of coated and uncoated concrete surfaces protected with four different coating materials: water-soluble (BW), solvent-based (BR), mineral (MI), and epoxy (EP). The durability assessment includes evaluating the absorption rate of water, pull-off adhesion strength, and coating material thickness. Concrete samples were subjected to immersion in regular water and a 7% urea solution, followed by cyclic freezing and thawing. Furthermore, the diffusion of chloride ions in concrete was evaluated using the impressed voltage method, with the samples exposed to the aging process immersed in a 3.5% NaCl solution. The results indicate that EP and BW coatings were significantly affected by the presence of urea and freeze–thaw cycles, resulting in a 43% and 47% reduction in pull-off adhesion strength, respectively. Notably, the MI-coated concrete samples exposed to urea solution and the freeze–thaw cycles exhibited a significant reduction in the absorption rate due to the accumulation of crystals on the coating surface, resulting in reduced porosity of the material.

## Introduction

The durability of concrete structures embedded in soil is primarily influenced by the porosity of the concrete and the chemical properties of the soil^1^. Concrete porosity directly affects the transport of chlorides, sulfates, CO_2_, leading to steel corrosion, cracking, and a reduced life span of reinforced concrete structures^2,3^. Reinforced concrete structures face challenges in various regions worldwide due to unfavorable environmental conditions. Moreover, these structures are additionally affected by soil contamination, resulting from various human industries such as energy, transport, construction, mining, and others. This contamination, combined with the impacts of climate change, accelerates the deterioration of reinforced concrete^4,5^.

During the service life of concrete structures, damage occurs due to chemical attacks and aggressive environments. The combustion of fuels, resulting in sulfuric acid, along with hydrochloric acid, aluminum chloride, and calcium bisulfite, leads to rapid concrete deterioration^6^. The presence of salts, sulfates, and alkalis contributes to leaching, increasing porosity and weakening the concrete matrix^7^. Additionally, freeze–thaw cycles at different temperatures generate hydraulic pressure and subsequent expansion of the pores of the concrete, causing cracking, scaling, and crumbling^2,8^.

To mitigate the deterioration of concrete matrix and, thus, the corrosion of reinforcing steel, it is crucial to implement an adequate protection technique. According to Aguirre-Guerrero et al.^9^, various systems can be employed to protect concrete structures, including improved structural design, incorporation of mineral additives, and application of coating materials. Implementing effective protection measures reduces permeability and extends the lifespan of structures^10^.

Coating materials can be applied to the reinforcing steel or the concrete surface. According to Taylor et al.^11,12^, categorized coatings into three groups: metallic, organic, and inorganic. Epoxy, water-soluble, acrylics, vinyl resin, urethane resins, and bitumen, are commonly used to protect concrete structures in the construction sector.^13,14^. The primary objective of these materials is to prevent the entry of chemical agents that could compromise the structural integrity of the concrete elements.

A. Almusallam et al.^15^ evaluated the adhesion strength, chloride permeability, and thermal variations of five types of coating materials, including epoxy resin. The results showed that the epoxy material exhibited the highest adhesion strength values when applied in one layer, achieving 3.3 MPa after two weeks of application. Overall, epoxy resin demonstrated excellent crack bridging ability, resistance to thermal variations, and low chloride permeability, outperforming polyurethane and acrylic coatings. In a similar study, Diamanti et al.^16^ investigated the effect of a modified cementitious coating exposed to regular water and chlorides. Adhesion strength, water absorption, chloride diffusion, and chloride penetration rate were assessed after two months of application. The study highlighted that this coating material significantly reduced the water content of concrete and improved its performance, particularly with an increased polymer/cement ratio, effectively reducing steel corrosion. Epoxy coatings have also been studied for their effectiveness in protecting against sulfates attacks^17^ and carbonation^18,19^.

Freeze–thaw cycles are a common natural phenomenon that can significantly impact the durability and performance of various materials, especially in regions with seasonal temperature fluctuations. The repeated freezing and thawing of water within the pores of materials causes the water to expand and contract, exerting substantial stresses on the material matrix. The severity and frequency of these stresses are determined by the temperature range at which the cycles occur^20^.

In particular, temperatures between −5 and + 15 °C have been identified as an optimal range for studying freeze–thaw effects. This range encompasses the transition phase from freezing to thawing and represents conditions commonly encountered in temperate climates. According to a study by Wang et al.^21^, approximately 60% of research papers use temperature ranges between −5 and + 15 °C in their freeze–thaw cycles.

By investigating freeze–thaw cycles within this temperature range, researchers can gain insights into the mechanisms and consequences of these cycles. They can also assess the performance and durability of materials, and develop strategies to enhance material resilience in environments prone to such cyclic conditions^22,23^.

The fatigue resistance and viscosity of modified bitumen coatings were evaluated under the influence of freeze–thaw cycles.. Tengfei et al.^8^ subjected the material to up to 18 cycles and used Fourier transform infrared (FTIR) to analyze the chemical composition changes. The results showed that the fatigue resistance decreased gradually after the freeze–thaw cycles, and the viscosity was excessively reduced. However, no changes were observed in the spectral peak range of 4000–2000 cm^−1^.

Four different coatings were used to protect concrete samples in this study. The samples were subjected to a unique accelerated aging method that involved freeze–thaw cycles, exposure to regular water and urea, and exposure to chlorides. Concrete samples without coating protection were used as reference samples. It is important to note that there is no published data on the results of the pull-off test and the impressed voltage technique using the proposed aging technique and materials.

The objective of this research study is to comprehensively evaluate the performance and behavior of four distinct coating materials utilized in the construction industry to safeguard concrete structures. The coatings will be subjected to exposure to two specific contaminants, namely water and urea, which are commonly encountered in real-world scenarios. The investigation will involve applying these protective materials to the surfaces of both reinforced and non-reinforced concrete samples.

To conduct this research, a set of carefully prepared concrete specimens will be utilized. These specimens will be divided into two groups: reinforced and non-reinforced concrete. Reinforced concrete includes additional materials such as steel bars, while non-reinforced concrete consists solely of cement, aggregates, and water.

Four distinct coating materials will be selected based on their common usage in the construction sector. These coatings may vary in their composition, formulation, or application technique, representing a range of protective materials available in the market. By using multiple coatings, the research aims to capture a comprehensive understanding of their individual behaviors and performance characteristics.

The findings of this research will provide valuable insights into the performance and behavior of the four coating materials when exposed to water and urea contaminants. The results can inform construction industry professionals, engineers, and material manufacturers about the most effective protective coatings to employ in specific environmental conditions.

## Experimental procedure, materials, and methods

### Experimental procedure

**Figure 1:**
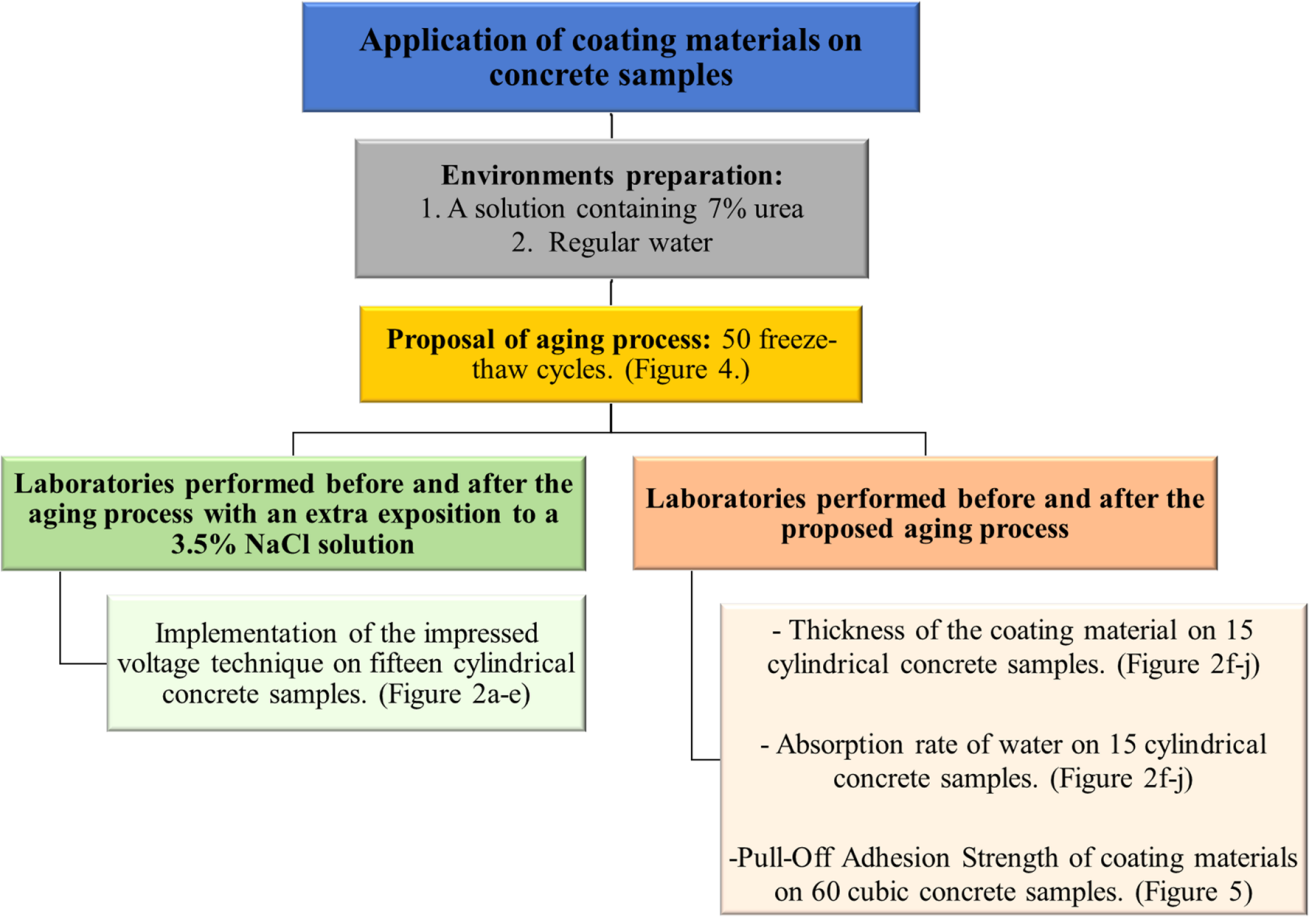
Research methodology.

This research study aims to evaluate the effectiveness of different coating materials applied to concrete surfaces exposed to various contaminants. The study will combine quantitative evidence of changes in adhesion strength, absorption, and coating thickness. The findings will contribute to the growing research area on concrete protection in contaminated environments. Figure 1 shows the research methodology used during this study.

### Materials

Four types of commercial coating materials commonly employed in the construction sector were used for the protection of reinforced concrete elements embedded in contaminated soil. These coating materials include water-soluble coating (BW), solvent-based coating (BR), mineral coating (MI), and epoxy coating (EP). Additionally, control samples without any coating material (NC) were evaluated. Table 1 describes the coating materials employed.

**Table 1:** Technical data of the coating materials.

Coating material	Composition	Main characteristics
Water-soluble coating (BW)	Industrial asphalt, organic solvent, additives, SBS	Bulk density: 1.05 ± 0.05 g/cm^3^ Water content: ≤ 50% (m/m)
Solvent-based coating (BR)	Water dispersion of asphalts, rubbers, and improvers	Water content: ≤ 0.5% (m/m)
Mineral coating (MI)	Polymer-modified and cement mixture	Relative density: 1.35 ± 0.05 g/cm^3^ Adhesion to concrete using the "pull-off" method: ≥ 3.0 MPa
Epoxy coating (EP)	[A] Epoxy resin based on bisphenol and [B] a polyamide hardener based on aliphatic polyamines	Compressive strength: ≥ 80 MPa Tensile strength: ≥ 25 MPa Bulk density after mixing: 1.10 g/cm^3^

The specifications of the concrete utilized for producing the specimens with dimensions of (100 × 100) mm are described as per the PN-EN 206+A2:2021-08 standard^24^ and PN-B-06265:2018-10 standard^25^. Table 2 provides a detailed description of the concrete utilized in this research.

**Table 2:** Concrete specifications.

Concrete properties	Description
Compressive strength class (test period 28 days)	20 MPa—type B20 (C16/20)
Type of cement	CEM II/B-V 32.5 R-HSR/NA
W/C ratio	0.77
Additions	Fly ashes
Consistency class	S3
Exposition class	X0
Aggregate dimension (Dmax)	16 mm
Class of chlorine content	Cl 0.40
Reaction to fire class	A1

The application process for the coating materials is described as follows:

- Firstly, the concrete surface was thoroughly cleaned using a regular brush to remove any dirt or debris after a curing period of 28 days.
- Before applying the MI coating, the concrete surface was properly moistened. This procedure minimizes water absorption of the mixing water by the concrete and reduces the potential for cracking of the mineral coating^26^
- The application of the coating materials was carried out in accordance with the specifications provided by the manufacturer. The protected samples were left to dry for approximately five days at room temperature of 20  ± 1 °C.
- For the preparation of the, a water/cement ratio of 0.20 was used.
- The epoxy material was mixed in a weight ratio of 4:1 [A: B]. The two components were thoroughly mixed using a plastic knife for a duration of 3 min to achieve a uniform consistency.
- The number of coating layers applied varied depending on the type of coating. One layer was applied for EP and MI coatings, while two layers were applied for BR and BW coatings.

**Figure 2:**
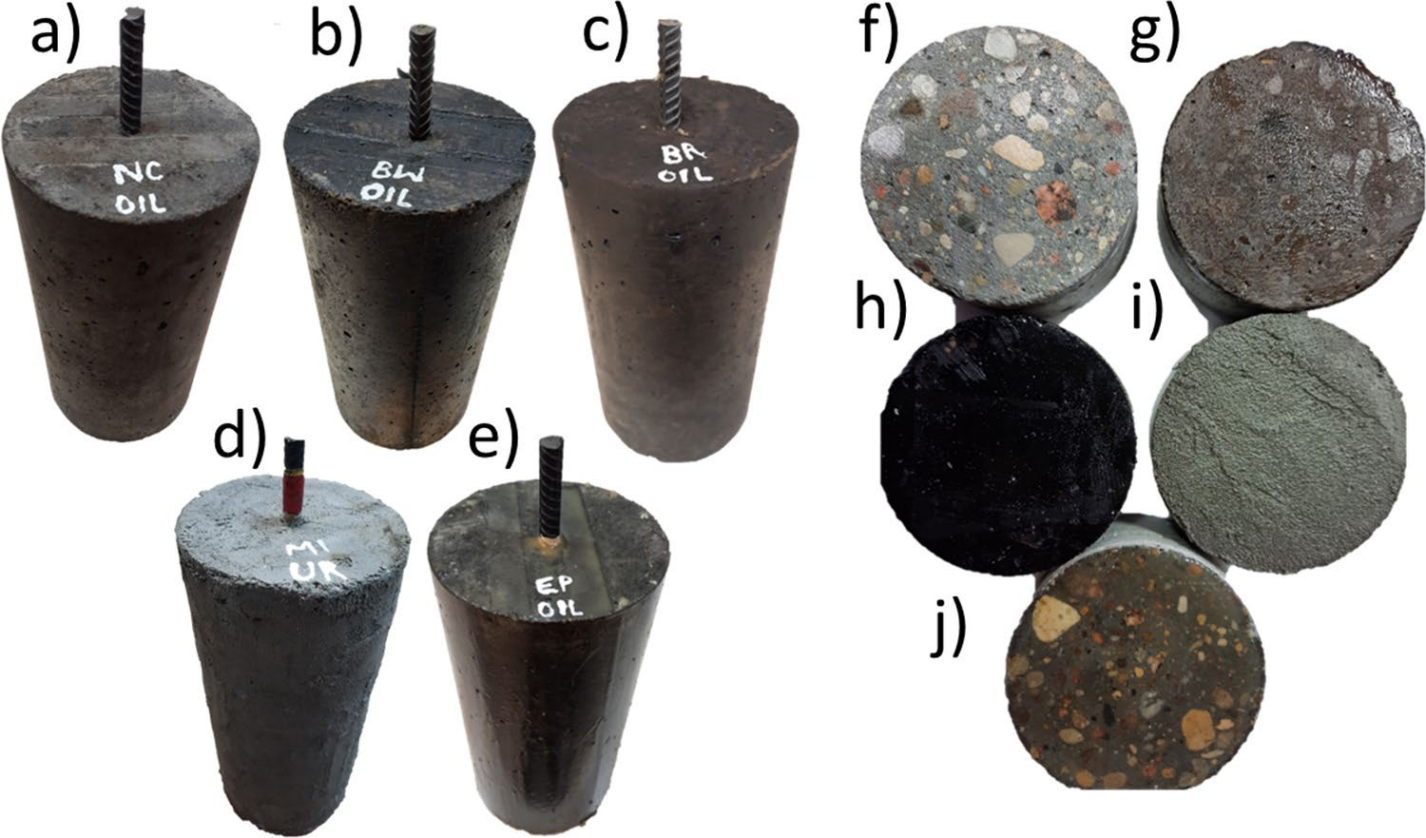
Reinforced concrete samples for the impressed voltage technique: (**a**) EP, (**b**) MI, (**c**) BW, (**d**) NC, (**e**) BR. Concrete samples for water absorption rate: (**f**) NC, (**g**) BR, (**h**) BW, (**i**) MI, (**j**) EP.

In this study three types of concrete samples were utilized. Pull-off adhesion strength tests were performed on cube-shaped specimens measuring 100 × 100 mm. Cylinders with reinforced concrete measuring 150 × 300 mm were used for the impressed voltage technique. Additionally, cylinders measuring 100 × 50 mm were employed to assess water absorption rates. Detailed illustrations of these concrete specimens can be found in Figs. 2 and 5.

**Figure 3:**
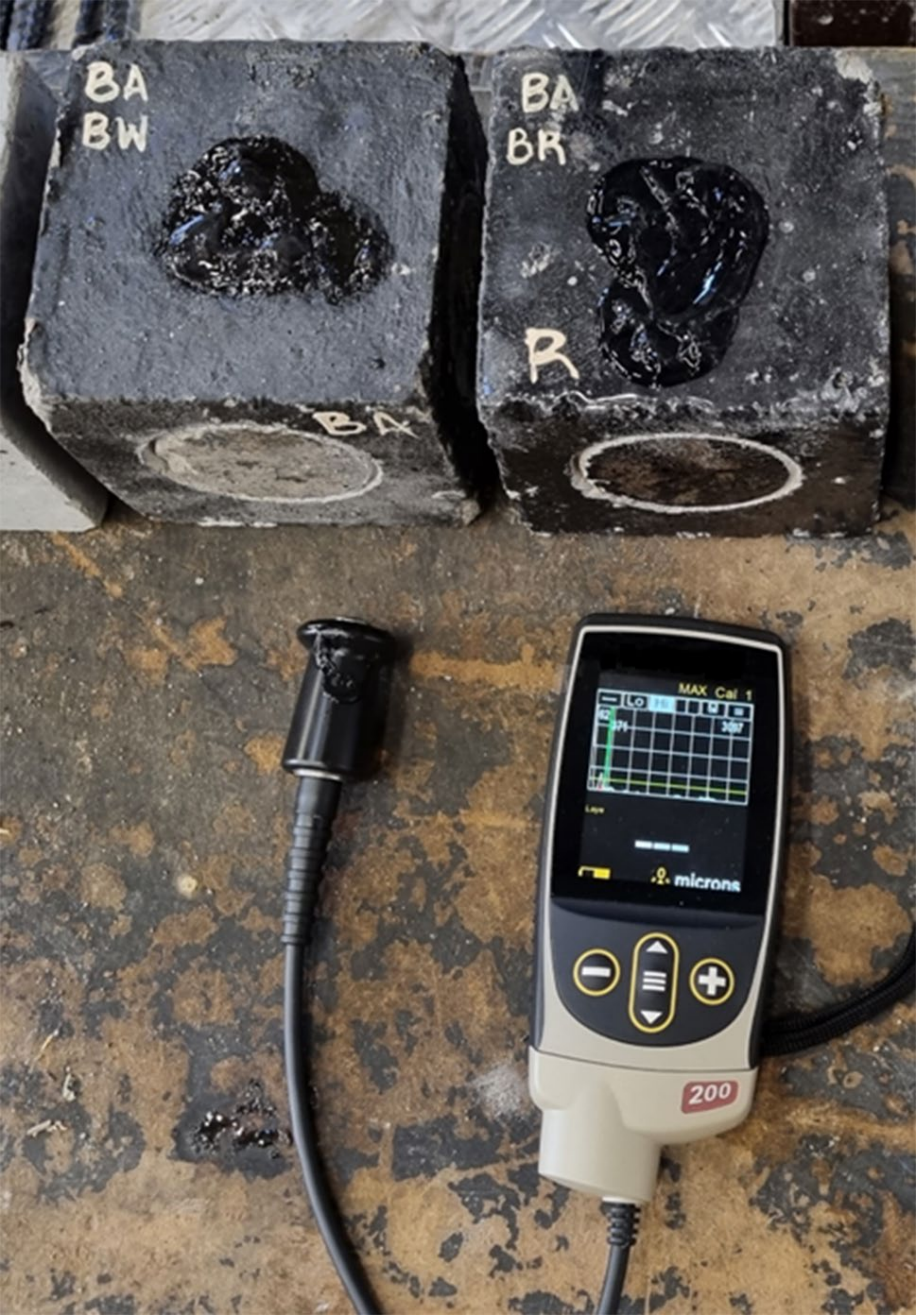
Measurement of coating material thickness.

Before and after the aging process and before the exposure of the specimens to saturation, the thickness of coating materials was determined using a PosiTector 200 coating thickness gauge. Figure 3 shows the equipment used for the thickness measurement.

## Experimental techniques

### Aging process

An accelerated aging test was conducted using a Toropol K-012 frost resistance test chamber, which is commonly employed for assessing the frost resistance of building materials. The specimens were subjected to cyclical freezing in air and thawing in water, while an electronic programmer monitored the cycles.

**Figure 4:**
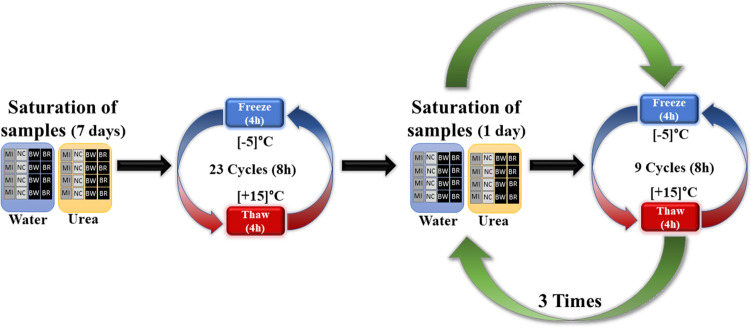
Aging process description. Freeze–thaw cycles and saturation of concrete samples.

To simulate freeze–thaw conditions in the laboratory, the test was performed at temperatures ranging between [−5] and [+ 15] °C. Two solutions were employed to simulate contaminated environments: regular water and urea solution with a concentration of 7%. The aging process, as depicted in Fig. 4, was applied to the samples. Furthermore, each specimen was coated with one of the four materials assessed in this research: BW, BR, MI, and EP. Additionally, a control sample without protection (NC) was included for comparison with the protected samples. After 50 freeze–thaw cycles and a 10-day saturation period, the absorption rate, pull-off adhesion strength, and chloride permeability of each sample were measured.

The aging process used in this research is a proper procedure proposed by our research team.

### Adhesion strength test

**Figure 5:**
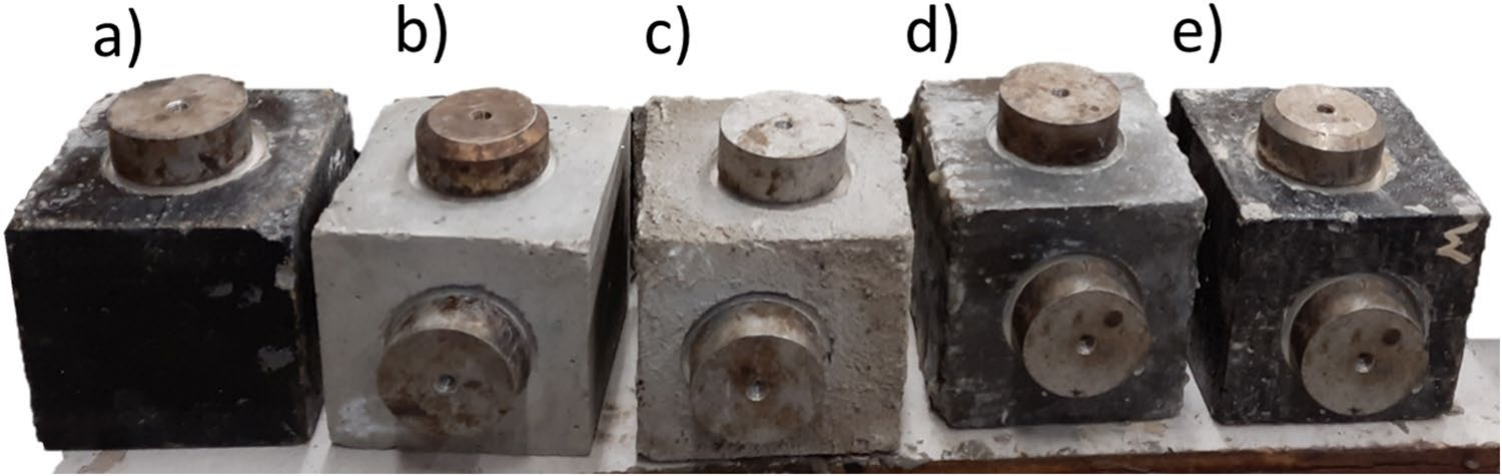
Concrete samples for the pull-off adhesion strength assessment (**a**) BR, (**b**) without coating, (**c**) MI, (**d**) EP, (**e**) BW.

The pull-off adhesion strength of coating applied to concrete surfaces was assessed in accordance with the ASTM D 7234-21 standard^4^. Cubic samples, as illustrated in Fig. 5, were used for the test.

To measure the adhesion strength, a DTH 500 DC testing machine with a maximum tensile strength of 5 kN was employed. Metallic dolls with a diameter of 50 mm and a height of 20 mm were utilized. To ensure accurate measurement, a circular notch was drilled to define the specific surface area to be measured. Subsequently, the metallic dolls were securely attached to the concrete surface using an epoxy material. Prior to the aging process, six pull-off tests were conducted for each coating material and environment type. The same procedure was repeated after the aging process.

### Absorption rate of water

The ASTM C1585-20 Standard^27^ was followed to determine the absorption rate of water. The coating materials were applied to only one side of the sample under controlled environmental conditions at 20 ± 2 °C and 65 ± 5% relative humidity. After curing for 7 days, the samples were dried in an environmental chamber at [+ 50] °C for 3 days. To ensure internal moisture equilibrium, the specimens were then stored in a sealed container for 15 days. Weight measurements were taken at various intervals, including up to 6 h and once daily for 8 days.

### Impressed voltage technique

To assess resistance to chloride ion permeability, the impressed voltage technique specified in the NT BUILD 356 standard^28^ was employed. A constant direct current of 5 V was applied between a steel bar embedded in the concrete sample and the concrete surface. Furthermore, reinforced concrete specimens were partially immersed in a 3.0% NaCl solution during the test duration.

**Figure 6:**
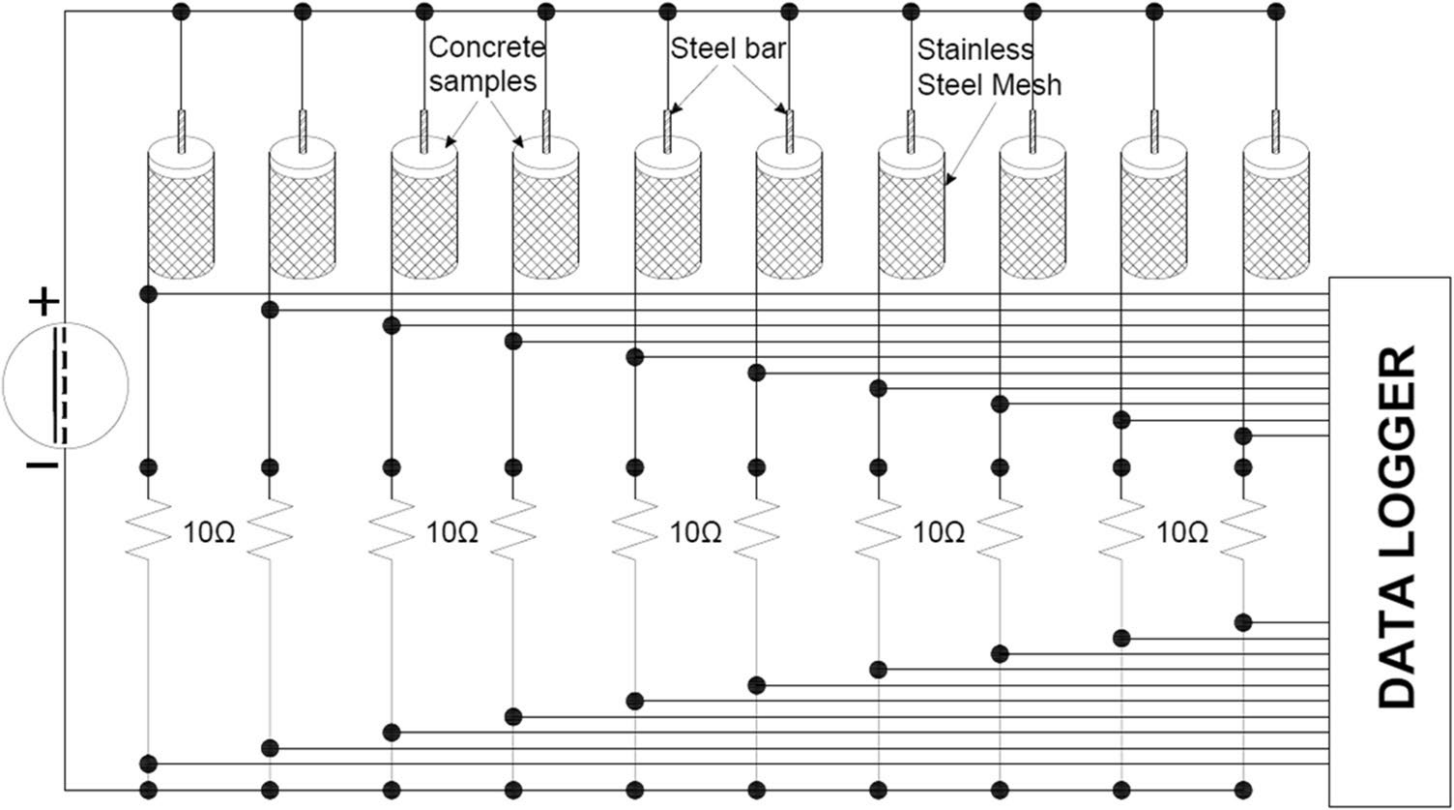
Electrical schematic design for the impressed voltage technique test.

This test was conducted both before initiating the aging process and after seven days of completing the aging process in both coated and uncoated reinforced concrete samples. The test concluded upon the appearance of cracks or corrosion on the surface of the steel bar. The circuit assembly utilized an external DC power supply, YIHUA 3005D [0–30 V] 5 A, and a measurement data acquisition system capable of connecting ten samples simultaneously. The schematic design is illustrated in Fig. 6.

## Results and discussion

### Pull-off adhesion strength test

**Figure 7:**
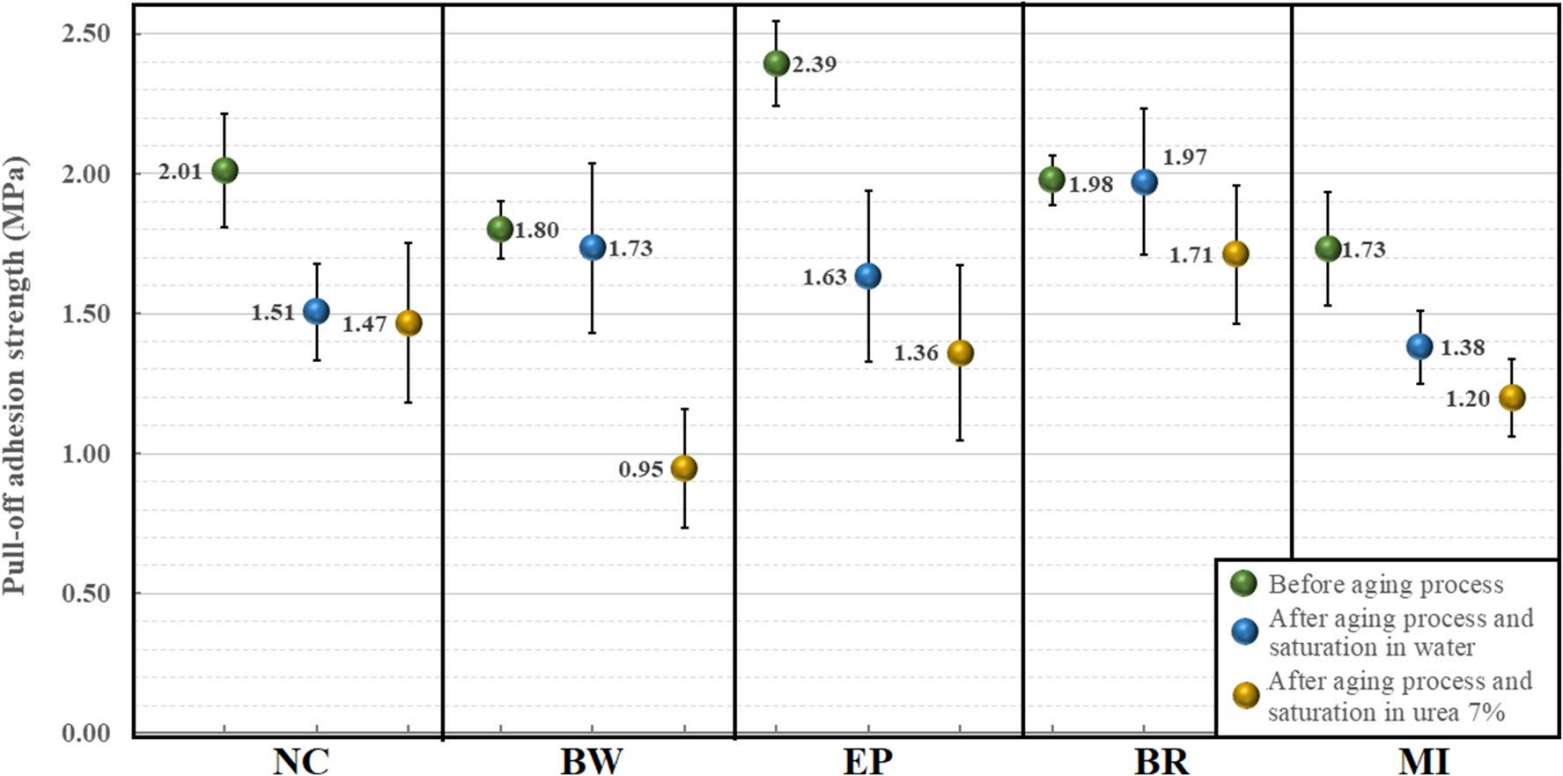
Average pull-off adhesion strength.

The pull-off adhesion strength results for concrete samples with and without coating material are depicted in Fig. 7. Adhesion strength values were calculated based on the average of six pull-off readings.

The results indicate that exposure to urea led to a decrease in the adhesion strength for all coatings after the aging process. Among the coatings, EP and BW were the materials more affected, exhibiting reductions of 43.09% and 47.22%, respectively, compared to the pre-aging process results. The obtained adhesion strength values for the EP coating, ranging between 1.36 and 2.39 MPa, align with those reported by A. Almusallam et al.^15^, who reported values within the range of 1.5 and 3.2 MPa. Similarly, the adhesion strength values for the MI coating align with the findings of Flores-Colen et al.^29^, who obtained values higher than 1.2 MPa for mineral and cement-based coatings. On the other hand, the BR coating exhibited the least reduction in adhesion strength after the aging process, with reductions of 0.51% for samples saturated in water and 13.63% for samples saturated in urea. This behavior can be attributed to the low water absorption of the material resulting from the presence of hydrocarbon constituents^30^.

In the case of EP coatings exposed to freezing temperatures, it is important to note that epoxy materials can undergo increased rigidity and become more vulnerable to fractures when subjected to stress^31^. This heightened susceptibility to damage during freeze–thaw cycles can be attributed to the sensitivity of epoxy materials to moisture. The cycling of freezing and thawing can introduce additional moisture into the epoxy, particularly if there exist microcracks or pores within the material. The presence of moisture has the potential to weaken the mechanical properties of the epoxy, leading to degradation or failure and ultimately resulting in a reduction in adhesion strength^32^.

The reduction in adhesion strength observed in BW coatings can be attributed to the formation of ice crystals during the freeze–thaw cycle. The repeated ingress of water followed by freezing can result in an increased moisture content within the material, rendering it more susceptible to damage. The expansion and growth of these ice crystals have the potential to disrupt the integrity of the bituminous material, ultimately leading to cracking and degradation^33^.

In contrast, solvent-based coatings (BR) are generally less affected by freeze–thaw cycles compared to other types of materials. This is primarily due to their low water content, which significantly reduces the formation of ice crystals. Solvent-based coatings exhibit better flexibility and toughness compared to water-based materials, allowing them to withstand the stresses and strains associated with freezing and thawing cycles. Their increased resilience and resistance to cracking or delamination contribute to their ability to withstand freeze–thaw conditions^34^.

Finally, even though mineral coatings usually are more susceptible to damage from freeze–thaw cycles compared to other types of coatings due to its high porosity and the different coefficients of thermal expansion of its materials, in our case the presence of modified polymers in the mixture improved the performance and resistance of MI coating, by incorporating polymers, such as acrylics, latexes, or styrene-butadiene rubber (SBR), into the mix, the resulting composite exhibits improved resistance to cracking^35^. The polymers can accommodate minor movements and stresses within the material, reducing the likelihood of crack formation and propagation, especially during freeze–thaw cycles or structural movement^36^. Additionally, it improves the adhesion to substrates and the bond strength and the most important the polymers create a denser and more water-resistant matrix, preventing water from entering the material and reducing the potential for freeze–thaw damage^37,38^.

Furthermore, the concrete samples without coating materials also experienced a reduction in concrete surface resistivity as a result of contaminants infiltrating the concrete matrix and exposure to freezing and thawing cycles. Similar results were reported by Penttala^39^ when concrete samples exposed to freeze–thaw cycles in saline and non-saline environments presented surface scaling and internal damages. According to Penttala, this deterioration is primarily attributed to the requirement for higher air content in low-strength concretes.

Additionally, to categorize the results, the four types of failures resulting from the pull-off test were determined in accordance with the ASTM D 7234-21^40^:

i. Glue failure [A]: Failure occurring between the epoxy used to attach the dollies to the concrete surface and the coating material.
ii. Cohesive failure [B]: Failure observed within the coating layer itself.
iii. Adhesive failure [C]: Failure that occurs between the coating material and the concrete surface.
iv. Substrate failure [D]: Failure identified when the adhesion of the coating is stronger than the tensile strength of the concrete surface.

**Figure 8:**
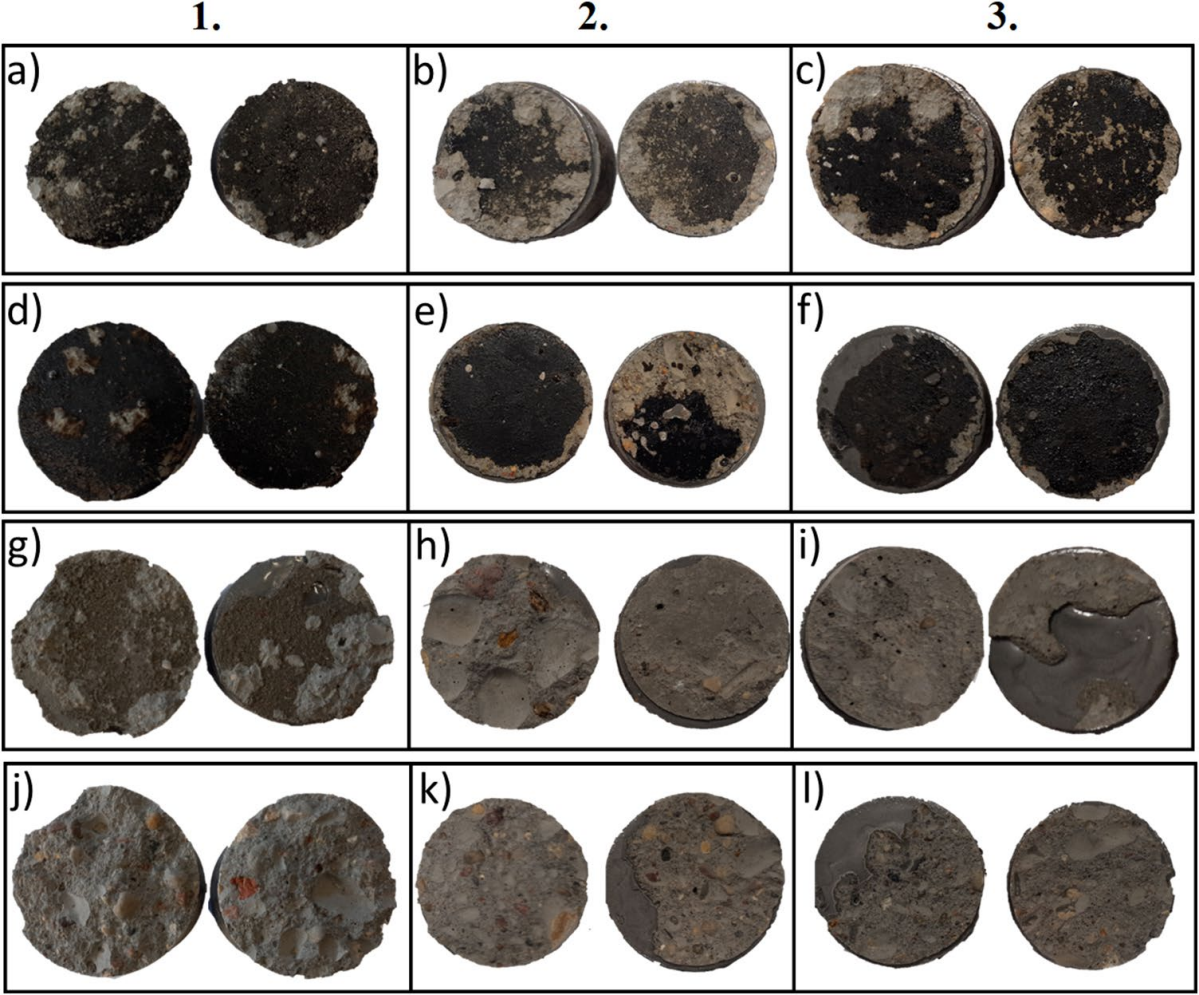
Pull-off adhesion failure types. (1) Samples before the aging process, (2) samples after the aging process and exposure to water, (3) samples after the aging process and exposure to urea—(**a**–**c**) BW, (**d**–**f**) BR, (**g**–**i**) MI, (**j**–**l**) EP.

Figure 8 showcases some examples of the failures encountered during this study. Column 1 presents the samples before the aging process, column 2 shows the samples after the aging process and exposure to water, and column 3 presents samples after the aging process and exposure to urea. In accordance with the failure types defined in ASTM D 7234-21^40^, all types of coatings exhibit adhesive failure [C] after the aging process and exposure to urea due. This can be attributed to the high contamination levels on the concrete surface caused by the urea components and the aging effects. Substrate failure, as depicted in (j), (k), (l), and (h), is the preferred mode of failure for coatings on concrete^40,41^. However, it is important to note that the cases of substrate failure observed in Fig. 8b, c for BW cannot be considered valid due to their low adhesion values.

A physical examination was conducted on the samples to analyze the structural changes in the concrete samples protected with the various coating materials.

**Figure 9:**
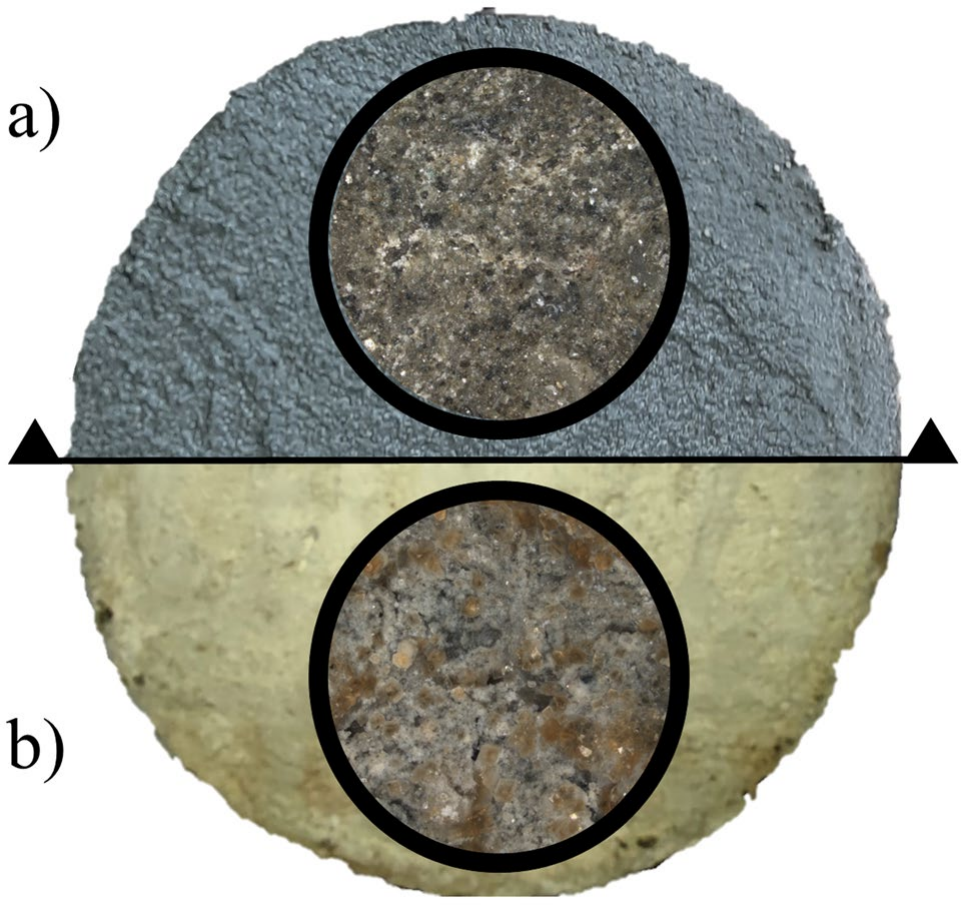
MI micrographs. (**a**) Before the aging process, (**b**) after the aging process.

For the MI coating, a noticeable accumulation of calcite crystal was observed after the aging process. This phenomenon can be attributed to the crystallization of urea on the concrete surface. similar results were reported by Ramakrishnan1 et al.^42^ in a study on the development of a self-repairing material able to remedy cracks and fissures in concrete using Bacillus pasteurii, Sporosarcina bacteria, and urea as a curing method. The study revealed the formation of calcite crystals accumulations on superficial cracks, which aided in filling them and reducing the infiltration of contaminants deeper into the concrete. Micrographs of the MI coating before and after the aging process with urea exposure are presented in Fig. 9, captured at a magnification of 10× (200 µm) under white light, clearly displaying the accumulation of calcite crystals.

However, in the case of BW and BR coatings, physical deterioration of the material was evident. Tengfei et al.^8^ reported that bitumen materials exposed to freeze–thaw cycles undergo changes in the content of different chemical compounds, leading to reduced durability of the material and adversely affecting the final adhesion strength values.

### Water absorption rate

**Figure 10:**
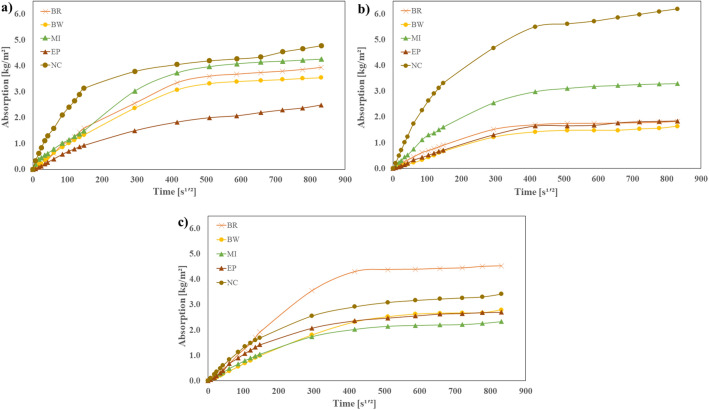
Water absorption rate (**a**) before the aging process, (**b**) after the aging process and saturation in water, (**c**) after the aging process and saturation in urea, 7%.

The results of water absorption rate for both coated and uncoated concrete samples before and after the aging process are illustrated in Fig. 10. All results comply with the ASTM C1585-20 Standard^27^, where a linear relationship for the initial and secondary absorption was observed, with a correlation coefficient higher than 0.98. Figure 10a shows the absorption rate behavior of samples before the aging process, which aligns with a study conducted by Millan et al.^43^. In their research, similar coating specimens were exposed to salt and regular water to assess their absorption properties. based on these findings, the MI coating exhibited the highest water absorption rate, followed by BR, BW, and EP coatings.

Figure 10b, c present the results of samples saturated with water and urea, respectively, and subsequently exposed to the aging process. These specimens presented a significant reduction in the absorption rate, primarily attributed to the aging process. The absorption rate of samples exposed to urea and protected with MI coating decreased due to the accumulation of salts in the coating surface, resulting in reduced permeability of the protected concrete. Conversely, for BR and BW coatings, a noticeable reduction in the absorption rate was observed for samples saturated with water and urea. It is evident that the exposure to urea deteriorates the coatings, leading to an increased absorption rate of 146.73% for EP, 247.54% for BR, and 171.16% for BW compared to samples immersed in regular water.

Table 3 presents the average thickness values of all coatings before and after the aging process, taking into account the accumulation of calcite crystal on the sample surface. After exposure to water and urea, a reduction in thickness weas observed, with BW experiencing the greatest drop of 57.78% for samples in water and 61.69% for samples in urea. Among the coatings, EP demonstrated one of the better performances, presenting reductions of 23.51% in water and 34.45% in urea.

**Table 3:** Average coating thickness for samples tested for absorption, adhesion strength, and impressed voltage technique.

Coating material type	Aging process stage	Average thickness depth (µm)
Water-soluble coating (BW)	Before aging process	343.00 ± 30.30
	After the aging process in water	144.80 ± 20.67
	After the aging process in urea (7%)	131.40 ± 19.78
Solvent-based coating (BR)	Before aging process	168.00 ± 20.44
	After the aging process in water	112.60 ± 5.02
	After the aging process in urea (7%)	146.00 ± 23.90
Mineral coating (MI)	Before aging process	534.00 ± 44.09
	After the aging process in water	532.00 ± 48.57
	After the aging process in urea (7%)	534.00 ± 23.68
Epoxy coating (EP)	Before aging process	415.00 ± 41.35
	After the aging process in water	317.40 ± 47.39
	After the aging process in urea (7%)	272.00 ± 45.09

The findings of the study reveal a compelling observation regarding the impact of aging processes on the thickness of coating materials. In particular, when subjected to both water and urea saturation, a discernible decrease in the coating material thickness becomes apparent. This reduction in thickness can have significant consequences for the overall properties of the coating, potentially resulting in a loss of color and compromised quality^44^.

The aging process of coating materials involves exposure to various environmental factors, including moisture and chemical substances like urea. When the coatings are subjected to water saturation, the moisture can gradually penetrate the material, causing it to undergo physical changes. The water may seep into the coating, leading to a swelling effect and subsequently causing the material to expand. Over time, this expansion can result in a reduction in the thickness of the coating layer as the material becomes more porous or undergoes partial dissolution^45^.

### Impressed voltage technique

**Figure 11:**
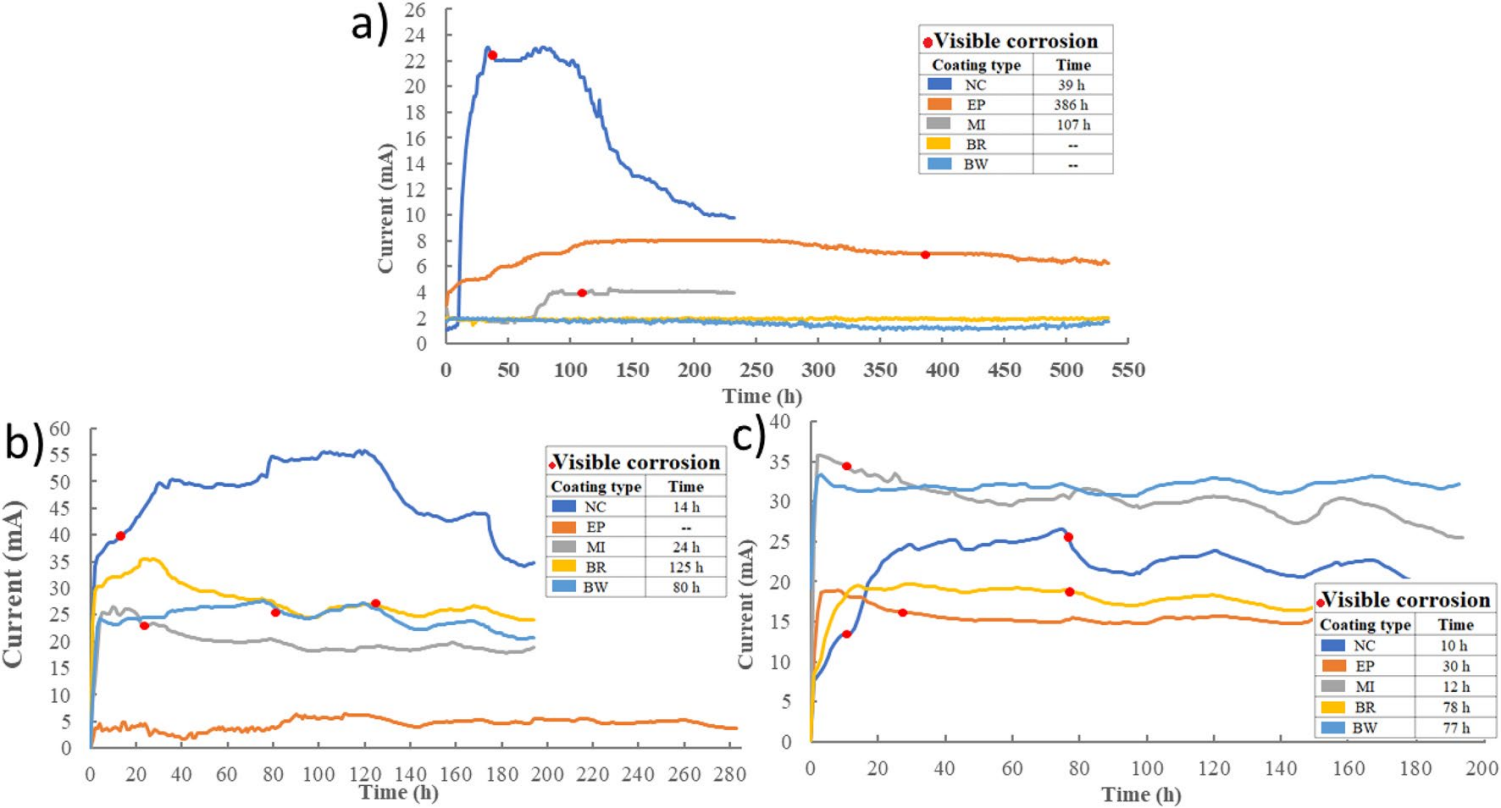
Current intensity curve, (**a**) before the aging process, (**b**) after the aging process and saturation in water, (**c**) after the aging process and saturation in urea, 7%.

The current intensity curves for samples before and after freeze–thaw cycles, saturated with regular water and urea, are presented in Fig. 11. Prior to the aging process, the maximum current for samples without coating protection was 23 mA, which was 58.18% lower than the samples saturated with regular water (55 mA). This increase indicates that the resistivity of the concrete surface has been compromised after the aging process, allowing for increased penetration of chloride ions into the concrete matrix. On the other hand, samples without coating protection and saturated with urea exhibited a current intensity 52.36% lower than samples saturated with water. This can be attributed to the presence of urea residues on the surface, which partially permeabilize the material and reduce the passe of chloride ions. Ramakrishnan1 et al.^42^.

MI, BR, and BW were the most affected materials by the freeze–thaw cycles. Before the aging process, these samples registered current values of 4.28, 2.08, and 1.98 mA, respectively. These values increased to 26.41, 35.54, and 27.73 mA for samples saturated in regular water and 35.78, 19.72, and 33.37 mA for samples saturated in urea solution. in the case samples protected with EP coatings, the highest current value of 18.93 mA was obtained in the samples exposed to urea, showing an increase of 235.10% before freeze–thaw cycles and 297.57% for samples saturated in water. These results align with previous findings by with Aguirre et al.^46^, where the highest current value for samples with epoxy coating was reported as 8 mA. Furthermore, it can be observed that, for all samples, the use of coating materials extended the appearance of corrosion products, thus protecting the concrete matrix in a chloride ion environment.

**Figure 12:**
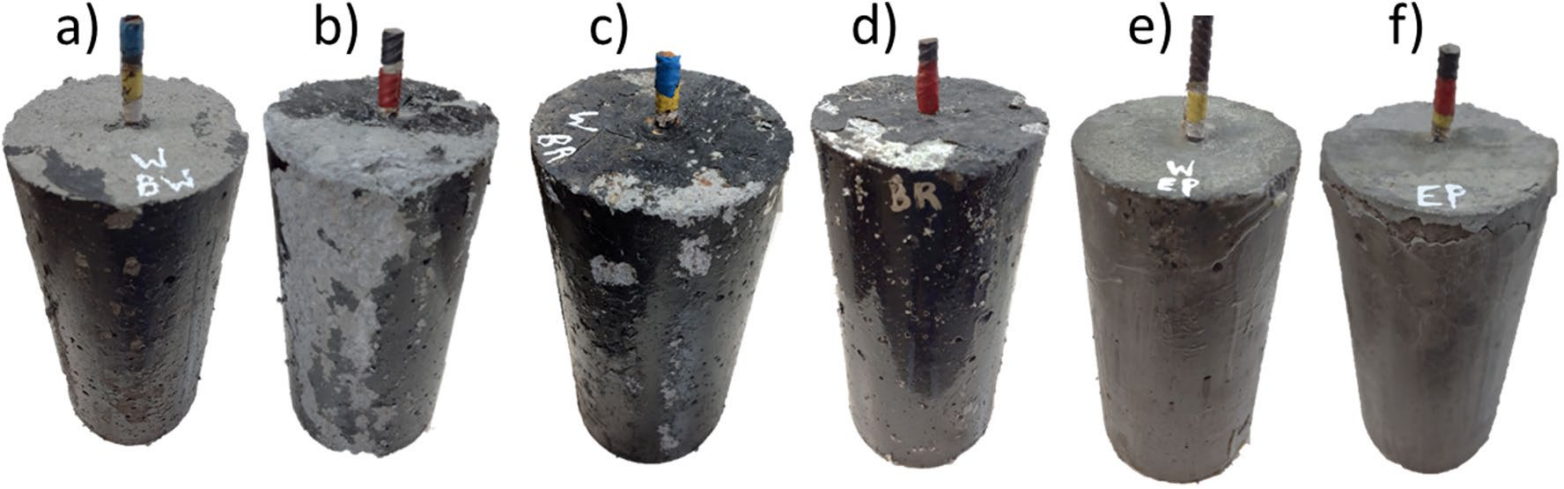
Concrete samples after the aging process. (**a**) BW water saturation, (**b**) BW urea saturation, (**c**) BR water saturation, (**d**) BR urea saturation, (**e**) EP urea saturation, (**f**) EP water saturation.

Figure 12 presents EP, BR, and BW concrete samples after the aging process, revealing visible physical damage in the coating materials. It is important to note that MI coating remained intact and undamaged throughout the aging process, regardless of exposure to water and urea. Similarly, EP samples exposed to water did not present any physical damage, effectively hindering the diffusion of chlorides into the concrete. However, EP samples exposed to urea, as well as BR, and BW samples exposed to water and urea, showed signs of physical damage. Among them, BW samples saturated with urea were the most adversely affected after the aging process.

The implementation of the impressed voltage technique in this study provides supplementary evidence that reinforces the relationship between absorption rate and coating degradation. The observed increase in current intensity, for all samples in both water and urea, and the accelerated appearance of corrosion products indicate a more rapid corrosion process when the absorption rate is higher. This conclusion is further reinforced by the substantial reduction in the time required for corrosion products to manifest. Specifically, the duration decreased from 386 to 30 h for EP, from 107 to 12 h for MI, and corrosion appearance was evident for BR and BW coatings after 77 h of exposure to the urea solution.

**Figure 13:**
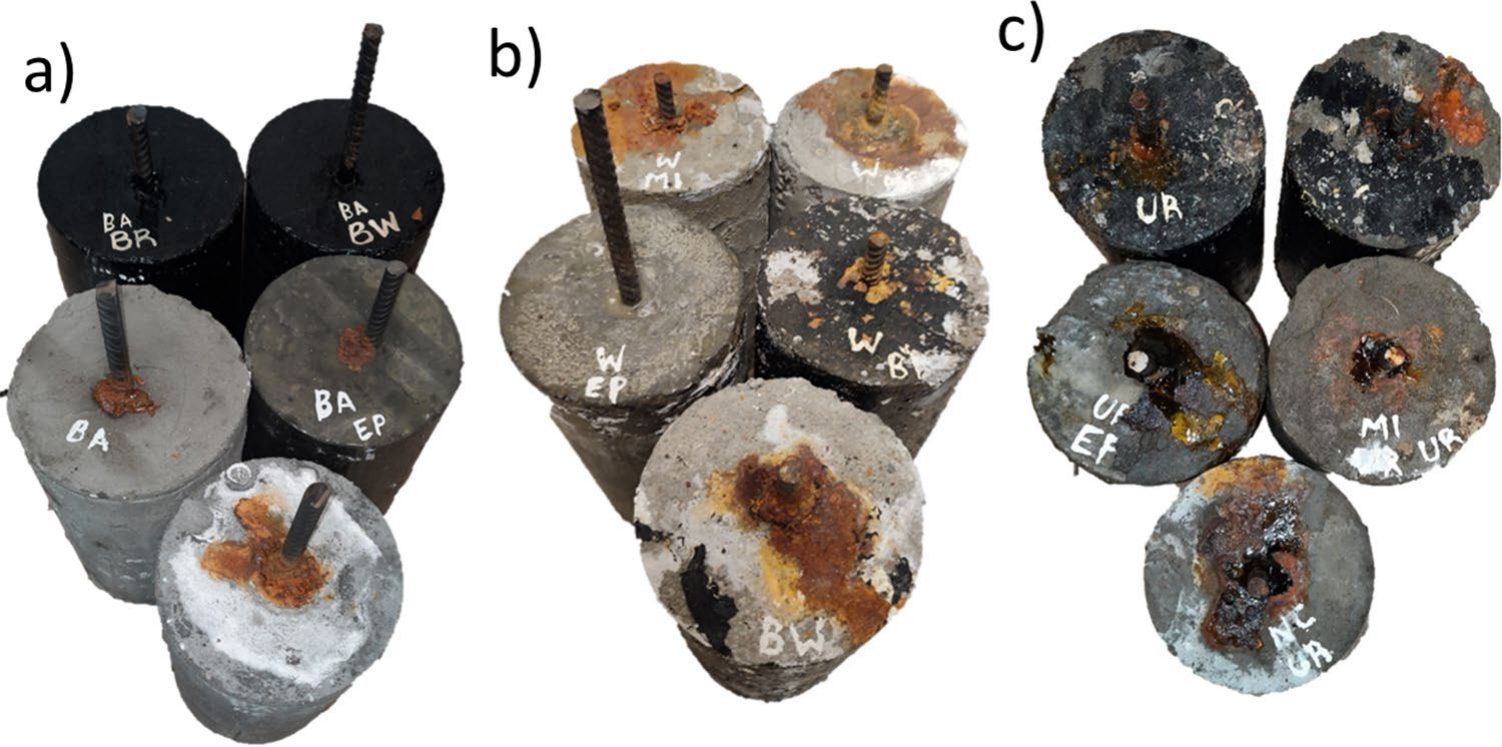
Concrete samples after the impressed voltage technique (**a**) before the aging process, (**b**) after the aging process and saturation in water, (**c**) after the aging process and saturation in urea, 7%.

Figure 13 presents the coated and uncoated reinforced concrete samples after the aging process and corrosion test. None of the specimens presented cracks on the concrete surface during the corrosion test, indicating that all laboratories were concluded once corrosion products became visible. After 22 days of testing, BW and BR samples did not present any corrosion on the concrete surface. In this case, the test was finalized, analyzing the materials' resistance to chloride ions. It is important to highlight that EP samples exposed to regular water did not present any physical damage before the impressed voltage technique. However, the presence of chlorides and the current that passes through the concrete deteriorated the coating material, leading to its fracture and physical damage.

## Conclusions

The pull-off adhesion strength of the different coatings presented a reduction after the freeze–thaw cycles. However, the presence of urea during the aging process led to a significant decrease in adhesion values, particularly for EP and BW coatings. The thickness measurements for these materials showed reductions of 34.46% and 61.69%, respectively, which likely contributed to the decrease in adhesion strength along with surface contamination by urea crystallization. In contrast, BR coating demonstrated a reduction of 13.10% in thickness and 13.63% in adhesion strength when exposed to urea solution, indicating it superior performance in terms of adhesion and thickness measurements after the aging process.

The results of absorption rate analysis revealed that MI, EP, and BW coatings, when exposed to regular water and urea, as well as BR coating exposed to regular water, exhibited a decrease in water absorption rate compared to samples that were not exposed to freeze–thaw cycles. Although these results were unexpected considering the reductions observed in adhesion strength and thickness, the decrease can be attributed to the accumulation of crystals on the coating surface. Moreover, the physical examination analysis indicating the formation of calcite crystals on the concrete surface when cured in the urea solution. These crystals effectively filled superficial cracks, thereby reducing the material's permeability.

The impressed voltage technique demonstrated an increase in current intensity after the freeze–thaw cycles. Furthermore, samples saturated with the urea solution exhibited higher current values compared to those saturated with regular water. However, regardless of the saturation solution, all types of coatings extended the appearance of corrosion products, with BR and BW coatings performing the best in this regard.

The findings of this study provide valuable insights into the behavior of epoxy-based coatings under aging conditions, particularly in relation to exposure to urea. The results clearly indicate that the adhesion strength of the epoxy coating is significantly reduced after undergoing the aging process, with urea exposure being a particularly influential factor in this degradation. The results demonstrate a direct correlation between the increase in the current intensity and the weakening of bonds between the coating and the substrate in the case of exposition to urea solution.

Overall, the findings indicate a decrease in adhesion strength and an increase in current intensity for all samples when exposed to regular water and urea. However, it is worth noting that there is no clear evidence of an increase in the absorption rate in the case or regular water. On the contrary, the results suggest that the absorption rate actually decreases with the aging process and water exposure. Further investigation is necessary to fully understand and elucidate this behavior.

A noticeable reduction in coating material thickness after the aging process, whether through water or urea saturation, has implications for the visual appearance and protective qualities of the coating. To mitigate these issues, further research and development may be necessary to enhance the durability and resistance of coating materials against aging processes. The formulation of coatings could be optimized to withstand prolonged exposure to water, urea, and other environmental stressors.

Based on the findings related to adhesion strength and absorption rate, the epoxy-based (EP) coating demonstrates its superiority as the optimal choice for providing effective protection to concrete structures. The results of this study provide substantial evidence supporting the suitability of EP coatings in safeguarding against deterioration and corrosion processes.

The consistently high adhesion strength exhibited by the EP coating, even after the aging process, suggests its ability to maintain a robust bond with the substrate. This strong adhesion is crucial for ensuring long-term durability and protection, as it minimizes the potential for coating detachment or delamination. The ability of the EP coating to withstand the adverse effects of aging, including exposure to urea and regular water, further validates its suitability for concrete protection.

### Research limitations

This paper was limited to English articles, which excludes literature published in other languages and is also limited to academic publications. Moreover, it does not consider the results from industrial practice.

## Data Availability

All data generated or analyzed during this study that support the findings are available from the corresponding author upon reasonable request and with the permission of all authors.
